# Reporting of angiographic studies in patients diagnosed with a cerebral arteriovenous malformation: a systematic review

**DOI:** 10.12688/f1000research.139256.1

**Published:** 2023-09-29

**Authors:** Suparna Das, Paul Kasher, Mueez Waqar, Adrian Parry-Jones, Hiren Patel

**Affiliations:** 1 The University of Manchester, Manchester, England, UK

**Keywords:** cerebral arteriovenous malformation, angioarchitecture, reporting, consensus

## Abstract

A cerebral arteriovenous malformation (cAVM) is an abnormal tangle of cerebral blood vessels. The consensus document by the Joint Writing Group (JWG) highlighted which cAVM features should be recorded. Subsequent publications have reported cAVM angioarchitecture, but it is unknown if all followed the JWG recommendations.

The aim of this systematic review was to describe use of the JWG guidelines.

A database search, using the PRISMA checklist, was performed. We describe the proportion of publications that used JWG reporting standards, which standards were used, whether the definitions used differed from the JWG, or if any additional angiographic features were reported.

Out of 4306 articles identified, 105 were selected, and a further 114 from other sources.

Thirty-three studies (33/219; 15%) specifically referred to using JWG standards.

Since the JWG publication, few studies have used their standards to report cAVMs. This implies that the angioarchitecture of cAVMs are not routinely fully described.

## Introduction

Cerebral arteriovenous malformations (cAVMs) cause death and disability mostly in the young. ^1^ The fact that cAVM treatment is associated with significant morbidity and mortality poses additional challenges. Treatment is currently aimed at shrinking or excising the lesion, usually after it has ruptured or caused symptoms. Management decisions require a case-by-case multidisciplinary discussion balancing risks and benefits: there is no definitive algorithm for management.

Typically, digital subtraction angiography (DSA) is used to classify cAVMs, which have a complex morphology with each malformation being unique. This makes reliably classifying cAVMs challenging for clinicians managing them. Although reliability studies have shown good intra-observer agreement on the characterisation of cAVM angioarchitecture, inter-observer agreement was poor. ^2^

To address this, a consensus document was published by the Joint Working Group (JWG) of the Technology Assessment Committee: this provides elementary and clear definitions of terms and recommends which clinical and radiological cAVM features should be described and recorded ( Table 1). ^3^ This group, consisting of neuroradiologists, neurosurgeons, stroke and interventional neurologists practising in the United States of America, was created to produce guidelines for cAVM research.

### Aims

The aim of this study was to systematically review the cAVM angioarchitecture literature to describe whether and how the JWG criteria were used.

## Methods

The review protocol was sent for registration to PROSPERO but not accepted due to “a perceived lack of direct impact on patient outcomes”. Reporting was in accordance with the Preferred Reporting Items for Systematic Reviews and Meta-Analyses (PRISMA) statement and checklist. ^4^

### Eligibility criteria

Peer-reviewed publications were searched from 1 Jan 2001 (when JWG standards were published) and limited to human subjects and the English language. There was no restriction on age or sex. Studies were excluded if they were reviews and/or exclusively discussed cavernous malformations, dural arteriovenous fistula, angioma, capillary telangiectasia, Vein of Galen Malformations or other angiographically occult vascular malformations.

### Information sources

A database search was performed using EMBASE and Medline, on 15/07/19 by one reviewing author (SD). It was repeated on 10/9/20 to update the search by a second author (MW). There was independent assessment by a librarian and another reviewing author (HP). In addition to the electronic searches, we conducted citation tracking, checked the reference lists, and reviewed the list of similar articles.

### Search strategy

To conduct searches of the Medline electronic bibliographic database, combinations of the following search terms were used.

Medical Subheadings: (Arteriovenous Malformations OR Arteriovenous Malformations, Intracranial) AND (Brain OR Intracranial)) AND (angioarchitecture OR angiogram OR angiographic OR aneurysm OR venous OR ectasia OR nidus OR angiogenesis OR varix).

Text Words: (Arteriovenous Malformations OR Arteriovenous Malformations, Intracranial) AND (Brain OR Intracranial)) AND (angioarchitecture OR angiogram OR angiographic OR aneurysm OR venous OR ectasia OR nidus).

### Study selection

**Figure 1.**
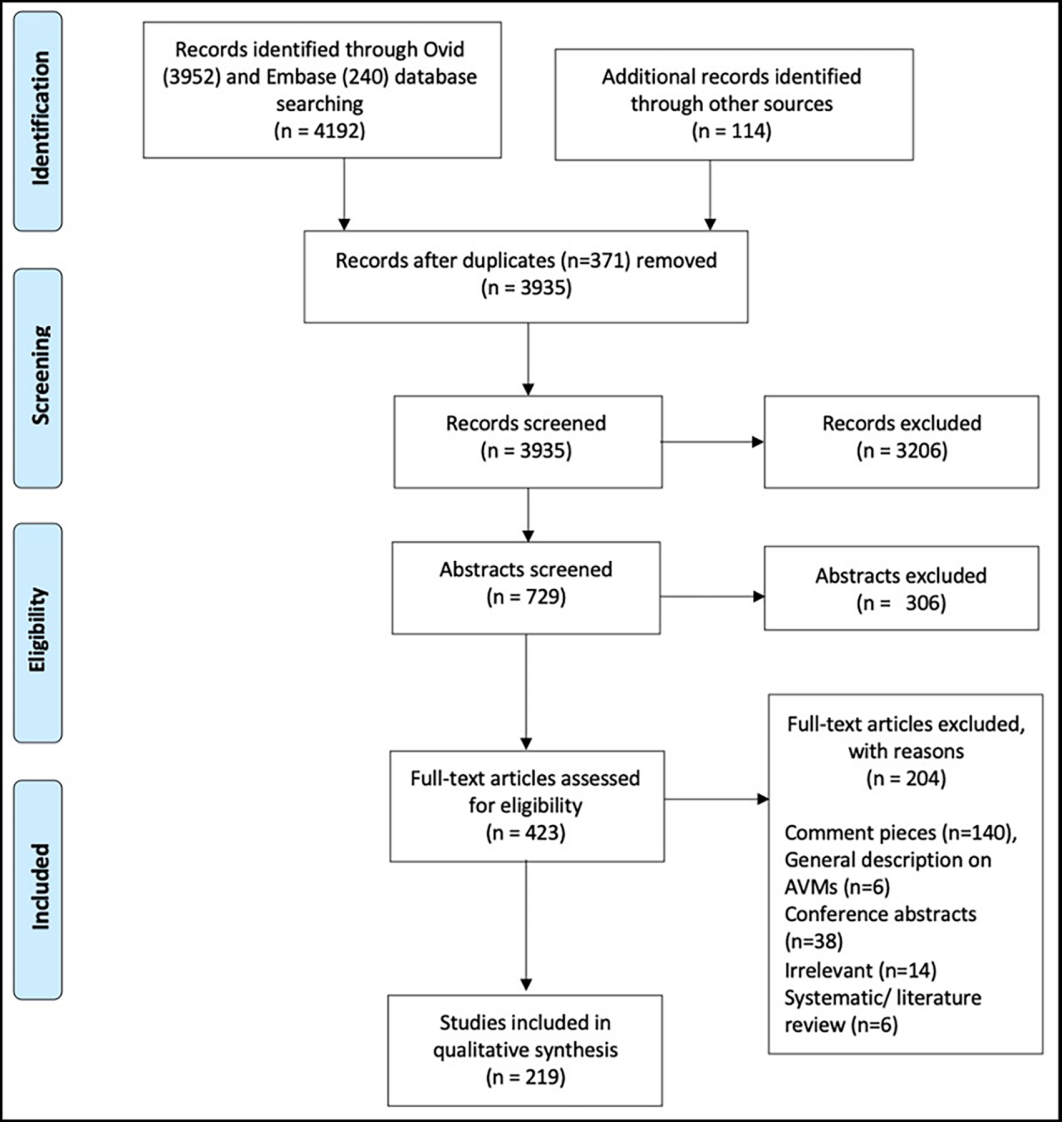
PRISMA flowchart demonstrates screening process for article selection.

Studies were selected if they included any of the search strategy’s features: titles and abstracts were reviewed. Figure 1 demonstrates how articles were excluded.

### Data collection process

Data extraction was performed by two independent reviewers (SD and HP) using the studies’ full text versions and by reviewing inclusion and exclusion criteria. Any disagreements were discussed between the two reviewers and an agreement reached. Pre-designed and piloted proforma were used.

### Data items

All the individual data items collected from each paper are listed in Table 1.

**Table 1.** The cAVM angioarchitecture fields suggested by the JWG. ^3^

	Definition/categorisation (if present)
Proposed fields	
• Location	• n/a
• Size	• recorded from MRI and angiography, measured in 3 dimensions (including cAVM’s largest diameter), volume calculated with ABC/2 formula
• Eloquence	• as per SMG score
• Border	• islands of normal cerebral parenchyma within cAVM nidus vs clearly outlined border with neighbouring parenchyma
Venous drainage	
• superficial vs deep	• Superficial: all cAVM drainage through cortical venous system. • Deep: if any/all drainage is through deep veins.
• periventricular	• drainage distinct from other deep venous drainage
• number of draining veins	• number of discrete venous channels leaving the nidus
• number of veins reaching sinus	• number of draining veins reaching any of these sinuses: superior sagittal, straight, transverse, sigmoid, cavernous, superior or inferior petrosal
• venous stenosis	• narrowing of any draining vein outflow pathway in two angiographic views
• venous ectasia	• more than double the calibre in any draining venous channel
• venous reflux	• flow reversal in any venous outflow pathway in a direction different than that to the nearest venous sinus
• sinus thrombosis	• dural venous sinus filling defect
Arterial supply	
• feeding arteries	• arterial structure which displays a flow contribution to the AV shunt on DSA
• arterial aneurysms	• parent feeding vessel’s saccular dilatations
• Moyamoya-type changes	• pattern of angiographic changes indicating feeding artery occlusion
• Pial-to-pial collateralisation	• recruiting of side-by-side pial-to-pial collaterals not part of the nidus
• Intravascular pressure measurements	• n/a

cAVM – cerebral arteriovenous malformation; SMG – Spetzler-Martin Grade; AV – arteriovenous; DSA – digital subtraction angiography.

### Risk of bias in individual studies

Risk of bias was determined by two independent reviewers (SD and HP) using a version of a score devised to determine the methodological quality of case series and case reports, ^5^ modified to expand its application to other study types. For each study, the quality assessment questions used were:

• Diagnosis: Are diagnostic criteria (as defined) for cAVM clearly identified and met?
• Is the method of cerebral angiography described, including the arterial injections, views taken, and what structures are included in a standard view?
• Is the method of calibration described?
• Are the cerebral angiograms reported on by two blinded neuroradiologist(s)?
• Were the patients reported collected over a short period of time in sufficient numbers?
• Is intra-rater reliability reported for each publication?

### Summary measures

The principal summary measures were the number of studies following the JWG terminology and the angioarchitectural fields described.

### Synthesis of results

This review aimed to assess if the description of cAVM angioarchitecture is standardised according to the JWG criteria and to explore if there are additional features that could be used to describe cAVMs and added to the JWG reporting standards. Additional features could be required to describe cAVMs as shown by some studies. Our objectives were to describe:

1. which of the JWG reporting standards were used,
2. adherence to definitions used in the JWG document,
3. novel angiographic features not mentioned by the JWG, and
4. the profession and experience of those reporting cAVMs.

We also compared the inter-observer agreement of different studies for the common criteria studied.

### Risk of bias across studies

Publication and selective reporting biases were assessed.

## Results

### Study search

We identified 4306 publications ( Figure 1) and 219 articles were selected for full-text review, reporting the angiogram findings potentially for 54, 148 individuals in total.

### Study designs

Most studies were retrospective (63%), with the remainder being prospective (27.9%), case reports (2.7%), studies that were both prospective and retrospective (2.7%), and educational (3.7%).

### Publication trends

The studies spanned from 2001 to 2020 (the JWG report was published in 2001). The number of patients in each study ranged from 1 to 3923. The median was 120 (interquartile range: 30 to 278). The countries individually publishing the highest number of studies were China (15.5%) and the United States of America (15.1%). Studies from Western Europe, North America and Asia made up 26.5% of studies, with other countries (publishing fewer studies, i.e. one to four studies each) making up 69.9%.

Beijing Tiantan Hospital published more papers than any other centre, with data from this single centre contributing to 26 (74.3%) of the studies from China and 11.9% overall. Several studies used the same study populations, occasionally with a few more cases added due to an extension of study duration by a few years ( Table 2). Out of Beijing Tiantan’s 26 studies, the commonest author groups were Lv et al and Tong et al (five studies for each author group) with the same sample population used three times for each of these two author groups ( Table 2). The University of California (San Francisco) (18; 8.2%), University of Virginia (Charlottesville) (7; 3.2%), and Columbia University (New York) (6; 2.7%) were the most frequently publishing American institutions.

**Table 2.** Author groups with same or overlapping study populations, with the number of studies each, and the mean sample size for each group.

Author group with overlapping study populations	Number of studies (%)	Mean sample size
Beijing Tiantan Hospital		
Lv, Wu, Jiang, Yang, Li, Sun, Zhang ^58^^,^^173^^,^^175^	3 (1.4)	302
Ma, Kim, Chen, Wu, Ma, Su, Zhao ^56^^,^^57^	2 (0.9)	108
Tong, Wu, Lin, Cao, Zhao, Wang, Zhang, Zhao ^55^^,^^176^^,^^68^	3 (1.4)	225
The First Affiliated Hospital of Guangzhou Medical University		
Pan, Feng, Vinuela, He, Wu, Zhan ^60^^,^^102^	2 (0.9)	152
University of California, San Francisco		
Du, Dowd, Johnston, Young, Lawton ^90^^,^^178^	2 (0.9)	304
John Hopkins University, Baltimore		
Yang, Liu, Hung, Braileanu, Wang, Caplan, Colby, Coon, Huang ^67^^,^^81^	2 (0.9)	123
University of Virginia, Charlottesville		
Ding, Starke, Quigg, Yen, Xu, Przybylowski, Dodson, Sheehan ^69^^,^^179^^,^^180^^,^^181^	4 (1.8)	1400
University of Illinois, Urbana-Champaign		
Shakur, Valyi-Nagy, Amin-Hanjani, Ya qoub, Aletich, Charbel, Alaraj ^62^^,^^63^^,^^143^^,^^170^	3 (1.4)	80
Columbia University, New York		
Stapf, Mohr, Pile-Spellman, Sciacca, Hartmann, Schumacher, Mast ^62^^,^^63^^,^^143^^,^^170^	4 (1.8)	542
Hospital Lariboisiere, Paris		
Choi, Mast, Hartmann, Marshall, Stapf ^168^^,^^169^	2 (0.9) same study population as Stapf et al	735

### Topics reported

Given the clinical importance of predicting haemorrhage, the aim of 65 papers (29.7%) was to test for associations between angioarchitecture and risk of bleeding (including location). ^1^^,^^6^^–^^69^ Twelve studies tested for an association between angioarchitecture and risk of seizure. ^70^^–^^81^

Standard imaging was compared against novel imaging techniques in four studies, and pre-operative imaging was investigated in two studies. ^82^^–^^87^ Three studies assessed haemodynamics. ^28^^,^^88^^,^^89^

Eleven papers studied various grading scores. ^82^^,^^90^^–^^99^ The Spetzler-Martin Grade was the most commonly analysed, but others included the Spetzler-Ponce, Lawton-Young, and Pollock-Flickinger. Out of these 11 studies, seven assessed and proposed different grading systems. ^92^^–^^96^^,^^98^^,^^99^

Four papers assessed the reliability of different cAVM grading scales, and two studies assessed the reliability in describing cAVM angioarchitecture. ^2^^,^^82^^,^^90^^,^^91^^,^^97^^,^^100^ Agreement ranged from fair to excellent for both inter- and intra-rater comparisons.

Angioarchitectural characteristics were reported in association with treatments: embolisation, ^17^^,^^43^^,^^46^^,^^92^^,^^94^^,^^100^^–^^129^ surgery, ^6^^,^^86^^,^^101^^,^^110^^,^^111^^,^^113^^,^^114^^,^^117^^,^^130^^–^^145^ and stereotactic radiosurgery. ^44^^,^^74^^,^^88^^,^^51^^,^^104^^,^^110^^,^^113^^,^^114^^,^^117^^,^^120^^,^^131^^,^^136^^,^^146^^–^^152^

In those studies that assessed inter-observer agreement, the commonest criteria investigated were size, SMG, venous drainage, and arterial feeders. The lowest scores were 0.62, ^82^ 0.46, ^82^ 0.56, ^83^ and 0.6 ^86^ respectively. The highest scores were 0.98, ^153^ 0.96, ^154^ 0.89, ^155^ and 0.91 ^155^ respectively.

### Quality of studies

Overall, the quality of the reporting studies was poor, with several of the study quality criteria not fulfilled ( Table 3). Only 48 out of 219 studies (21.9%) used the definitions recommended by the JWG for some of the features reported ( Table 3), ^7^^,^^10^^,^^11^^,^^14^^,^^15^^,^^17^^,^^19^^–^^21^^,^^24^^,^^26^^,^^29^^,^^38^^,^^40^^,^^46^^–^^48^^,^^52^^,^^55^^,^^57^^,^^58^^,^^62^^,^^63^^,^^66^^,^^68^^,^^81^^,^^143^^,^^147^^,^^150^^,^^156^^–^^172^ with only 33 publications (15.1%) reporting and specifically mentioning using the JWG standards.

**Table 3.** Publications listed by authors in alphabetic order, associated with criteria assessing study quality. The list excludes studies which do not describe angioarchitecture in detail e.g. only report location. White boxes indicate criteria fulfilled; dark grey boxes indicate criteria absent. a = JWG standard used; b = cAVM diagnostic criteria; c = DSA method: arterial injections; d = DSA method: views; e = Calibration method; f = Inter-rater reliability assessed; g = Statistics performed.

Study author	a	b	c	d	e	f	g
Abla 2014 ^65^							
Abecassis ^185^							
Al-Shahi ^2^							
Al-Tamimi ^186^							
Alen ^187^							
Alexander ^7^							
Anderson ^188^							
Benson ^79^							
Bharatha ^189^							
Blanc ^128^							
Braileanu ^190^							
Brunozzi 2017 ^191^							
Brunozzi 2019 ^157^							
Burkhardt ^167^							
Chang ^192^							
Chen ^193^							
Choi 2006 ^168^							
Choi 2009 ^169^							
Chowdhury ^159^							
Cuong ^84^							
D'Aliberti ^36^							
De Blasi ^194^							
de Castro-Afonso ^195^							
Guo ^37^							
Halim 2004 ^30^							
Halim 2002 ^38^							
Hernesniemi ^202^							
Hetts ^203^							
Hofmeister ^164^							
Huang ^23^							
Hung 2019 ^40^							
Iancu-Gontard ^100^							
Illies ^16^							
Imbesi ^204^							
Iryo ^205^							
Jayaraman 2012 ^106^							
Jiang ^206^							
Jiao ^93^							
Jin ^15^							
Kakizawa ^207^							
Kandai ^10^							
Kellner ^8^							
Khaw ^20^							
Kim 2004 ^24^							
Kim 2007 ^41^							
Kim 2014 ^21^							
Kouznetsov ^208^							
Dinc 2019 ^33^							
Dinc 2018 ^196^							
Ding 2017 ^27^							
Ding 2015 ^80^							
Dos Santos ^161^							
Du 2005 ^197^							
Du 2016 ^198^							
Ellis ^11^							
Fierstra ^78^							
Fleetwood ^199^							
Fok ^14^							
Frisoli ^97^							
Fukuda 2016 ^200^							
Fukuda 2017 ^162^							
Fullerton ^47^							
Galletti ^76^							
Garcin ^163^							
Gauvrit ^83^							
Geibprasert ^201^							
Griessenauer ^91^							
Kubalek ^54^							
Kurita ^209^							
Lee ^210^							
Liew ^211^							
Lin ^212^							
Liu 2015 ^73^							
Luo 2012 ^174^							
Lv 2013 ^173^							
Lv 2011a ^175^							
Lv 2011b ^58^							
Lv 2015 ^213^							
Ma 2017a ^56^							
Ma 2017b ^57^							
Ma 2015 ^64^							
Majumdar ^48^							
Miyasaka ^31^							
Morgan 2016 ^131^							
Motebejane ^214^							
Neidert ^95^							
Nishino ^215^							
Nisson 2020 ^96^							
Niu ^13^							
Ognard ^82^							
Orning ^35^							
Oulasvirta ^216^							
Ozyurt ^153^							
Pan 2013 ^60^							
Patel ^217^							
Pawlikowska ^26^							
Pekmezci ^218^							
Reitz ^12^							
Riordan ^45^							
Robert 2014 ^219^							
Robert 2017 ^94^							
Robert 2015 ^127^							
Sahlein ^52^							
Schmidt ^39^							
Schwartz ^220^							
Shakur 2016a ^32^							
Shakur 2016b ^89^							
Shakur 2018 ^221^							
Shakur 2015 ^222^							
Shankar ^70^							
Sheng ^223^							
Shotar ^99^							
Singh ^85^							
Stapf 2003 ^170^							
Stapf 2002b ^171^							
Stapf 2006 ^63^							
Stefani 2001 ^59^							
Stefani 2002 ^61^							
Stein 2016b ^114^							
Stein 2015 ^172^							
Sturiale ^49^							
Suzuki ^87^							
Tanaka ^135^							
Taschner ^155^							
Tasic ^50^							
Todaka ^18^							
Togao 2019 ^224^							
Togao 2020 ^154^							
Tong 2016a ^176^							
Tong 2016b ^55^							
Tong 2016c ^68^							
Tritt ^225^							
Tsuchiya ^86^							
Unlu ^226^							
Wrede ^227^							
Yamada ^1^							
Yang 2016b ^29^							
Yang 2017 ^34^							
Yang 2016a ^67^							
Yang 2015b ^81^							
Yang 2015a ^166^							
Ye ^228^							
Yi ^158^							
Yu ^22^							
Zwanzger ^229^							

### Risk of bias within studies

Biases in the studies were because there was a small population size (less than 100 cases) in 100 studies, a second professional did not independently review angiograms in any of the studies, and there was often a re-analysis of datasets.

### Number of studies reporting individual angioarchitectural features

**Figure 2.**
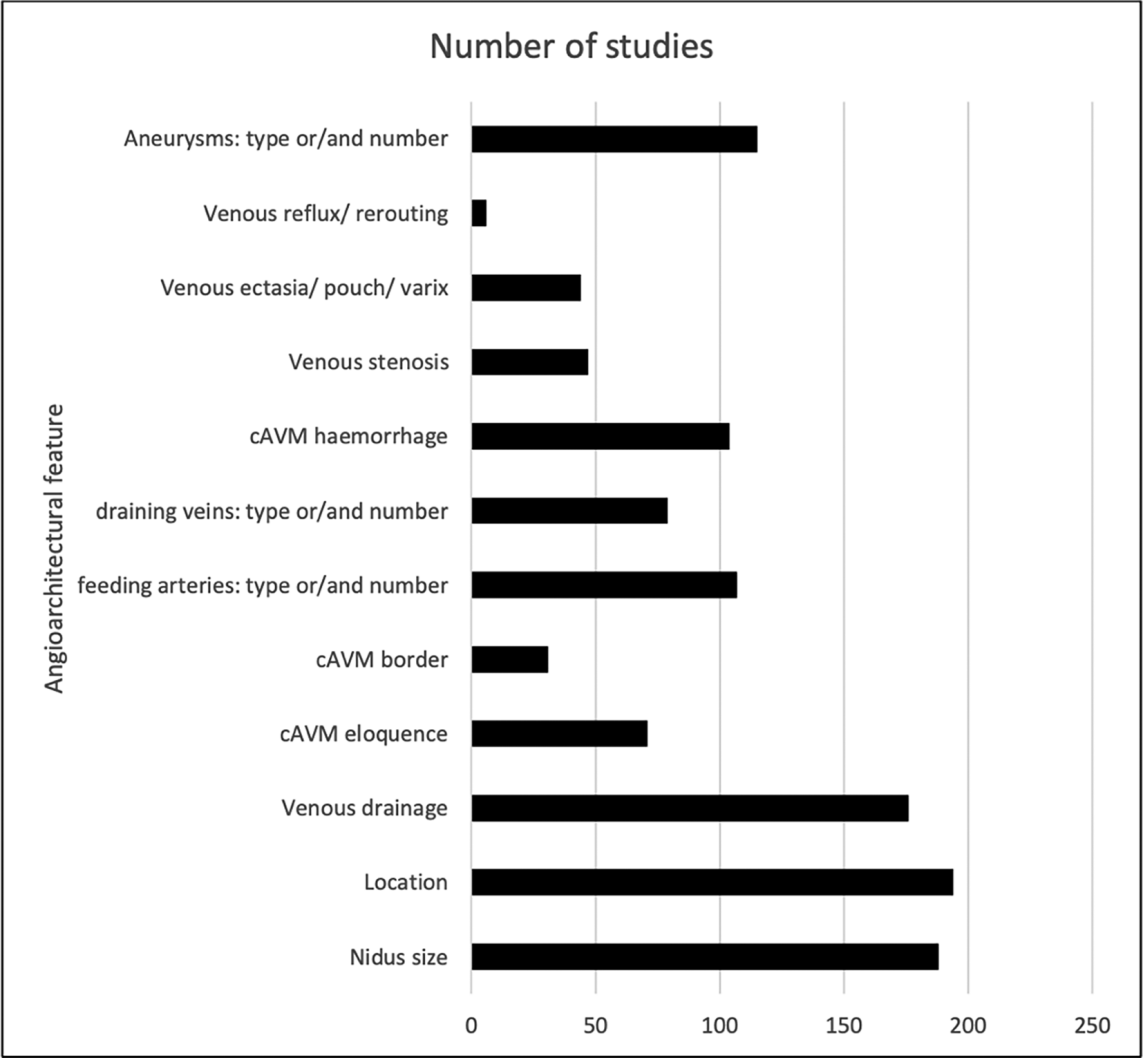
Key angiographic features listed in the Joint Writing Group’s recommendations, and the frequency with which there were reported on and defined in the studies identified.

The common angioarchitectural features are listed with the associated number of studies ( Figure 2). Most studies described nidus size (175 studies; 78%), location (153; 68%), border (29; 12.9%), venous drainage (173; 76.9%), feeding arteries (88; 39.1%), and the presence of aneurysms (121; 53.8%). No studies described the angiographic features of pial to pial collaterals or Moya-Moya type changes as recommended by the JWG.

Many studies used a variety of the recommended angiographic features, though not necessarily defining these features in the same way as the JWG ( Table 4).

**Table 4.** Angiographic features recommended for reporting cAVMs by the JWG associated with the number of studies that record each feature. They may have different definitions for these features compared to that stipulated by the JWG.

Angiographic feature described by the JWG	Number of studies (%)
**AVM size**	175 (78)
**AVM location**	153 (68)
**Eloquence**	72 (32)
**AVM border**	29 (12.9)
**Venous drainage**	173 (76.9)
**Number of draining veins**	71 (31.6)
**Venous stenosis**	47 (20.9)
**Venous ectasia**	42 (18.7)
**Feeding artery**	88 (39.1)
**Aneurysm**	121 (53.8)
**Moyamoya-type changes**	0
**Pial-to-pial collateralisation**	0
**Intravascular pressure measurements**	0

JWG: Joint Writing Group, cAVM: cerebral arteriovenous malformation.

Almost all the features described using the JWG guidelines were given different definitions, including, type of feeders, arterial feeders, and haemorrhagic presentation.

cAVM location was not described by the JWG, and this feature had the largest range of definitions. Some specified what constitutes deep, cortical, and/or infratentorial ^1^^,^^15^^,^^23^^,^^58^^,^^173^^,^^174^ or dichotomised location into supratentorial and infratentorial. ^20^^,^^138^^,^^175^^,^^176^ There were further categorisations into posterior fossa and periventricular by Ma *et al*. ^165^

Venous ectasia was the feature with the second-most variations of definition. Whereas the JWG defines it as double the calibre change in any draining venous channel, others have described it as 1.5 times larger than the contralateral vessel, ^177^ and two papers are broader in their definitions, describing venous ectasia as a markedly ectatic vein, ^98^ or an abnormal dilatation. ^59^^,^^61^

Aneurysms were defined as a saccular luminal dilatation of parent feeding vessels by the JWG. Most papers have essentially stated the aneurysm should be double the width of the artery, with only one definition stating the diameter is at least the same as that of the parent vessel. ^19^

Numerous studies described angioarchitectural features which were not mentioned in the JWG report and these are described in Table 5. These features included perinidal angiogenesis, AVM nidus, deep location, and venous varix/pouch.

**Table 5.** The most commonly described additional angiographic features (with their associated definitions) that are not listed in the JWG standards.

Angiographic feature	Definition	Number of studies
**AVM nidus**	• the vascular mass included in the AVM size measurement (Stapf 2003, Stapf 2006, Khaw) • the junction between the feeding arteries and draining veins, without a capillary bed (Mohr)	4
**Perinidal angiogenesis**	• Vascular network within brain parenchyma between the nidus and feeding artery terminal segment, without visible arteriovenous shunts (Valavanis) • Indirect supply to the AVM periphery from arterial branches other than the main arterial feeders (Shankar) • The formation of a new network of arteriocapillaries in the white matter around an AVM in reaction to hypoxia. This is caused by the steal effect from a high flow nidus in the perinidal brain (Taeshineetanakul, Hu)	4
**Deep location**	• Includes basal ganglia, internal capsule, thalamus, and corpus callosum (Lin) • The larger portion of the nidus is localised in deep white matter tracts, basal ganglia and thalamus, peri-ventricular regions, or posterior fossa (da Costa 2009) • Includes the cerebellum, thalamus, basal ganglia, internal capsule, corpus callosum, and brainstem (Hu)	3
**Venous varix/pouch/ectasia**	• Markedly ectatic vein (Lv 2013, Luo) • Focal dilatations at least twice as large as the vein diameter (Pan 2013) • Focal aneurysmal dilation in the draining venous system (Daou) • Proximal draining vein’s focal aneurysmal dilation (Chen 2017) • Bleb that originates on the nidus venules with no defined relationship with a draining vein (D’Aliberti) • Change of greater than 200% in the focal venous diameter of any drainage vein (Hu)	7

JWG: Joint Writing Group, cAVM: cerebral arteriovenous malformation.

### Professions conducting studies

The most common profession conducting these studies were neuroradiologists/neuro-interventionalists (101 studies) and neurosurgeons (60 studies). A neuropathologist was involved in one study.

## Discussion

We have shown that only 33 studies of 219 (15.1%) included in our systematic review explicitly followed the JWG standards since their publication 20 years ago. ^3^ Additionally, most studies reported venous drainage (76.9%), cAVM size (78%), and cAVM location (68%), suggesting these features are frequently considered as cAVM angioarchitecture. These parameters were the most widely used, likely due to their relation to the SMG system.

Since 219 publications were reviewed as providing data on angioarchitecture, it appears that this topic is considered important. Most commonly angioarchitectural features were used to test for associations with outcomes relevant to cAVM such as haemorrhage. Given that overall, the technical quality of publications was low, that most studies were retrospective and from small single centre series, the validity of results from these series could be questioned. Data reported from larger series also lacked the full consideration of angioarchitecture and often the same dataset was used for association studies again compromising the associations reported. In addition, as a large proportion of studies were published by single institutions, the results may not be generalisable to other cAVM populations.

In those studies that assessed inter-observer agreement, the criteria most frequently used for comparison were size, SMG, venous drainage, and arterial feeders. It is possible that the other criteria were less used as they were more difficult to analyse on imaging.

Twenty years have passed since the publication of the JWG definitions and, not unsurprisingly, several papers have reported on additional aspects of angioarchitecture which the JWG had not considered. These additional features may be helpful in understanding cAVMs and consideration should be given for their inclusion in any future update. These features are perinidal angiogenesis, deep location, venous and arterial dilatation. Angiogenesis is important for the formation and development of a cAVM and its presence may be useful in surgical planning. ^126^ The precise location of a cAVM is crucial with well accepted definitions key for a shared understanding when discussing patient management. Venous dilatation is helpful to describe as it indicates if there may be high or low-pressure flow in the cAVM, with a larger vein reducing the pressure in a cAVM. ^36^^,^^60^^,^^152^^,^^165^^,^^173^^,^^174^^,^^197^ This would be relevant to decide on the management approach. Equally, arterial dilatation ^20^^,^^77^^,^^151^^,^^177^ may imply high-pressure flow in a cAVM, particularly if combined with a single vein of regular dimensions and may have clinical implications.

In this review, we also observed that pial-pial collaterals and Moya-moya changes were not recorded. This may reflect the difficulty in identifying these features, but also may suggest that they occur infrequently.

## Conclusion

The JWG publication did clarify that the definitions were parameters to be used in research studies. ^3^ They have also discussed that there were no minimal criteria that should be used, emphasising that the angioarchitectural criteria were based on reasoned speculation. However, given that many of the criteria are likely to be interdependent, and studies are increasingly used to show associations with clinical presentation, this review would support the need to establish another working group to incorporate additional angiographic features and to include more specific and precise definitions for some of the features that were left open to interpretation. We would argue that these recommendations should then be widely publicised and uniformly incorporated into national and local reporting guidelines to help guide research and to ensure that clinicians can appropriately interpret this research with the understanding of the common language.

### Registration and protocol

This review was not registered as described above. The review protocol can be accessed on BioStudies. The link is https://www.ebi.ac.uk/biostudies/studies/S-BSST1168. The accession number is S-BSST1168.

## Data Availability

BioStudies. Review of angioarchitecture literature SD. DOI: https://www.ebi.ac.uk/biostudies/studies/S-BSST1168BioStudies. The raw data is in two files titled:
- ‘Review of angioarchitecture literature SD’ and - ‘Review of angioarchitecture literature quality SD’. The content is raw data from the papers used in the systematic review. The accession number is S-BSST1168. Biostudies. PRISMA checklist. DOI: https://www.ebi.ac.uk/biostudies/studies/S-BSST1168. The flow diagram is in Figure 1.
