# Supplementary material for: Reporting of angiographic studies in patients diagnosed with a cerebral arteriovenous malformation: a systematic review
Source: F1000Res. 2024 Nov 25;12:1252. Originally published 2023 Sep 29. [Version 3] doi: 10.12688/f1000research.139256.3 (PMC11809685; doi:10.12688/f1000research.139256.3)
Supplement: Peer review history

# Reviewer report: Reviewer response for version 3

**DOI:** 10.5256/f1000research.175125.r343194

**Published:** 2025-01-02

**Authors:** Mustafa Ismail

**Affiliations:** 1 Univeristy of Baghdad, Baghdad, Iraq

No further comments.

Are the rationale for, and objectives of, the Systematic Review clearly stated?

Partly

Is the statistical analysis and its interpretation appropriate?

Not applicable

If this is a Living Systematic Review, is the ‘living’ method appropriate and is the search schedule clearly defined and justified? (‘Living Systematic Review’ or a variation of this term should be included in the title.)

Yes

Are sufficient details of the methods and analysis provided to allow replication by others?

Yes

Are the conclusions drawn adequately supported by the results presented in the review?

Yes

Reviewer Expertise:

Neurosurgery

I confirm that I have read this submission and believe that I have an appropriate level of expertise to confirm that it is of an acceptable scientific standard.

# Reviewer report: Reviewer response for version 3

**DOI:** 10.5256/f1000research.175125.r343192

**Published:** 2024-12-31

**Authors:** Johan Wikström, Maria Correia de Verdier

**Affiliations:** 1 Uppsala University, Uppsala, Sweden; 2 Department of Radiology, Uppsala University, Uppsala, Sweden

The authors have responded to most of our concerns. I still see that cAVM location is described as NA in table 1, which I find inconsistent. Otherwise, I have no remaining issues.

Are the rationale for, and objectives of, the Systematic Review clearly stated?

Yes

Is the statistical analysis and its interpretation appropriate?

Not applicable

If this is a Living Systematic Review, is the ‘living’ method appropriate and is the search schedule clearly defined and justified? (‘Living Systematic Review’ or a variation of this term should be included in the title.)

Not applicable

Are sufficient details of the methods and analysis provided to allow replication by others?

Yes

Are the conclusions drawn adequately supported by the results presented in the review?

Yes

Reviewer Expertise:

MRI of the brain.

We confirm that we have read this submission and believe that we have an appropriate level of expertise to confirm that it is of an acceptable scientific standard.

# Reviewer report: Reviewer response for version 2

**DOI:** 10.5256/f1000research.169457.r335899

**Published:** 2024-11-15

**Authors:** Johan Wikström, Maria Correia de Verdier

**Affiliations:** 1 Uppsala University, Uppsala, Sweden; 2 Department of Radiology, Uppsala University, Uppsala, Sweden

This article aims to assess the adherence to the Joint Writing Group guidelines regarding description of features in brain arteriovenous anomalies. The authors have responded to previous reviewers´ comments. I have a few additional remarks;

 Why use "cAVM" and not "BAVM" as proposed by the JWG guidelines?

 In Methods, inclusion/exclusion criteria could be stated more clearly. Is DSA a requirement for inclusion?

 In table 1, regarding "location: NA", According to the JWL guidelines, there is a long list of locations to choose from.

 The listed items under " *Risk of bias in individual studies"* do not completely correspond to Table 3 and some of these results are not reported.

 In first sentence of Results, change "...219 articles were selected for full text review" to "...423 full-text articles were assessed for eligibility and 219 included in the study"

 On page 8, please describe what countries that "Western" papers originate from.

 On page 13, " *cAVM location was not described by the JWG," is not correct (see above).*

*The conclusion could be* clearer and better reflect the results and aim of this article.

Are the rationale for, and objectives of, the Systematic Review clearly stated?

Yes

Is the statistical analysis and its interpretation appropriate?

Not applicable

If this is a Living Systematic Review, is the ‘living’ method appropriate and is the search schedule clearly defined and justified? (‘Living Systematic Review’ or a variation of this term should be included in the title.)

Not applicable

Are sufficient details of the methods and analysis provided to allow replication by others?

Yes

Are the conclusions drawn adequately supported by the results presented in the review?

Yes

Reviewer Expertise:

MRI of the brain.

We confirm that we have read this submission and believe that we have an appropriate level of expertise to confirm that it is of an acceptable scientific standard, however we have significant reservations, as outlined above.

# Response

**Published:** 2024-11-20

**Authors:** Suparna Das

**Affiliations:** Neuroscience, The University of Manchester, Manchester, England, UK

Why use "cAVM" and not "BAVM" as proposed by the JWG guidelines?

 cAVM was the term used for my doctorate thesis, which this systematic review was part of.

 In Methods, inclusion/exclusion criteria could be stated more clearly. Is DSA a requirement for inclusion?

 The search terms are listed in the Methods section, and they include 'angiogram' So DSA is mentioned as an inclusion criteria.

 In table 1, regarding "location: NA", According to the JWL guidelines, there is a long list of locations to choose from.

 I have modified Table1 accordingly.

 The listed items under " *Risk of bias in individual studies"* do not completely correspond to Table 3 and some of these results are not reported.

 I have modified the paragraph and the table caption.

 In first sentence of Results, change "...219 articles were selected for full text review" to "...423 full-text articles were assessed for eligibility and 219 included in the study"

 I have made this change.

 On page 8, please describe what countries that "Western" papers originate from.

 The countries are now specified.

 On page 13, " *cAVM location was not described by the JWG," is not correct (see above).*

*I have amended this line.*

# Reviewer report: Reviewer response for version 1

**DOI:** 10.5256/f1000research.152519.r264835

**Published:** 2024-05-08

**Authors:** Mustafa Ismail

**Affiliations:** 1 Univeristy of Baghdad, Baghdad, Iraq

The authors reported a systematic review about "Reporting of angiographic studies in patients diagnosed with a cerebral arteriovenous malformation: a systematic review" and I congratulate them for this work. There are a few points to consider in this review:

  

1. **Enhancement of the Introduction:**
  - The introduction should be expanded to more clearly articulate the clinical implications of inconsistent reporting standards. Detailing the direct impacts on patient outcomes and treatment efficacy can better highlight the necessity for standardized reporting.
2. **Importance of Citing the JWG Standards:**
  - The article should more strongly emphasize the significance of the Joint Writing Group (JWG) standards in establishing a uniform framework for reporting. Additionally, it should address dissenting views regarding the sufficiency of these guidelines by presenting evidence from the review, demonstrating the clarity and comparability brought by adherence to JWG standards.
3. **Broadening the Discussion Section:**
  - The discussion should be expanded to incorporate a wider range of arguments regarding the variations in adherence to JWG standards across different medical practices or regions. It should analyze the implications for multi-center studies and longitudinal research and include perspectives from various stakeholders like clinicians, researchers, and healthcare policymakers.
4. **Inclusion of a Limitations Section:**
  - A dedicated section on limitations should be added to discuss both the methodological constraints of the review, such as potential publication bias, and the practical challenges of applying JWG standards universally, like variations in technological access across health systems.
5. **Rationale for Mandatory JWG Citations:**
  - The article should provide a robust justification for the inclusion of JWG citations in all related studies, explaining how these references enhance the academic rigor and credibility of research publications. It should also critique reasons given by some authors for omitting these standards, suggesting areas where JWG criteria could be expanded or adapted.

Are the rationale for, and objectives of, the Systematic Review clearly stated?

Partly

Is the statistical analysis and its interpretation appropriate?

Not applicable

If this is a Living Systematic Review, is the ‘living’ method appropriate and is the search schedule clearly defined and justified? (‘Living Systematic Review’ or a variation of this term should be included in the title.)

Yes

Are sufficient details of the methods and analysis provided to allow replication by others?

Yes

Are the conclusions drawn adequately supported by the results presented in the review?

Yes

Reviewer Expertise:

Vascular Neurosurgery, General Neurosurgery,

I confirm that I have read this submission and believe that I have an appropriate level of expertise to confirm that it is of an acceptable scientific standard, however I have significant reservations, as outlined above.

# Response

**Published:** 2024-07-20

**Authors:** Suparna Das

**Affiliations:** Neuroscience, The University of Manchester, Manchester, England, UK

We have addressed all the comments and we have modified mainly the introduction and discussion, but also some of the results.

# Reviewer report: Reviewer response for version 1

**DOI:** 10.5256/f1000research.152519.r211337

**Published:** 2023-11-02

**Authors:** Nitin Mukerji

**Affiliations:** 1 Department of Neurosurgery, South Tees Hospitals NHS Foundation Trust, Middlesbrough, England, UK

The authors have picked out a standard method of reporting/describing AVMs in literature and attempted to establish compliance via a retrospective review. While the work done is commendable, well researched and drafted appropriately; there are a few criticisms:

1. The reason why there is not much compliance with JWG guidelines is that every publication uses one of the available classifications, Spetzler-Martin, Spetzler-Ponce, Lawton-Young or Flickinger-Pollock - that is enough to give a basic idea of what one is dealing with and the granularity of the JWG is not always required.
2. With a lot of papers coming from one institution, in China - whether or not there is awareness of the JWG guidelines there is questionable - the conclusions are probably to be taken with some skepticism. It would help to know if the compliance was better in 'Western' papers.
3. Bias issues and tests such as 'reporting by two independent neuroradiologists' - I think are too harsh; this rarely, if ever happens.
4. I think this paper fundamentally is missing a 'premise' - what is it that the authors wish to convey? If the aim is to relate the fact that there is an obscure guideline most don't know about and hence nobody follows it - then; it is a wasted effort - most clinicians who deal with AVMs are aware of this. If the aim is to propose a new guideline and hence create the preamble that sufficient chaos exists in reporting - then that is not clear. The results and discussion needs to be written clearly to reflect this premise.
5. Sometimes the reason certain aspects are not reported ie. venous stenosis, ectasia...etc - is that they do not exist; that seems not to have been acknowledged.

Otherwise, a good manuscript - but the message needs to be clearer.

Are the rationale for, and objectives of, the Systematic Review clearly stated?

Yes

Is the statistical analysis and its interpretation appropriate?

Partly

Are sufficient details of the methods and analysis provided to allow replication by others?

Yes

Are the conclusions drawn adequately supported by the results presented in the review?

Partly

Reviewer Expertise:

Cerebrovascular Neurosurgery

I confirm that I have read this submission and believe that I have an appropriate level of expertise to confirm that it is of an acceptable scientific standard, however I have significant reservations, as outlined above.
